# The Auditory Processing Domains Questionnaire (APDQ): Brazilian–Portuguese version^☆^

**DOI:** 10.1016/j.bjorl.2021.12.001

**Published:** 2022-01-04

**Authors:** Karin Ziliotto Dias, Cynthia Harumi Yokoyama, Maria Madalena Canina Pinheiro, Joel de Braga Junior, Liliane Desgualdo Pereira, Brian O’Hara

**Affiliations:** a Universidade Federal de São Paulo (UNIFESP), Departamento de Fonoaudiologia, São Paulo, SP, Brazil; b Universidade Federal de São Paulo (UNIFESP), Departamento de Fonoaudiologia, Especialização em Audiologia, São Paulo, SP, Brazil; c Universidade Federal de Santa Catarina (UFSC), Departamento de Fonoaudiologia, Florianópolis, SC, Brazil; d Universidade Federal de Santa Catarina (UFSC), Curso de Pós-graduação em Fonoaudiologia, Florianópolis, SC, Brazil; e Developmental Behavioral Pediatrician, Honolulu, United States

**Keywords:** Auditory perception, Surveys and questionnaires, Hearing, Attention, Child

## Abstract

**Objective:**

The present study aimed to determine the fidelity, reliability, and internal validity of the APDQ when translated from English to Brazilian–Portuguese language.

**Methods:**

The first phase included the APDQ translation into Brazilian–Portuguese including forward translation, translation assessment and back-translation in a group of 10 students. The second phase involved establishing the internal consistency and reliability of the APDQ Brazilian–Portuguese version. Data was collected from 66 students without any risk factor for auditory processing disorder. Subjects were divided into a younger group (7–10 yrs) and an older group (11–17 yrs).

**Results:**

All items showed appropriate quality in terms of translation. In determining internal consistency, Cronbach's alpha in subscales of auditory processing, attention and language was found to be 0.93, 0.85 and 0.74, respectively. Intraclass Correlation Coefficient for total score was 0.95, ensuring a strong test-retest reliability.

**Conclusion:**

The Brazilian–Portuguese version of the APDQ has favorable translation quality, internal validity and reliability. It is now ready for continued study as a differential screening tool for 7- to 17-year-old Brazilian children with listening difficulties who are at risk for auditory processing disorder, attention deficits and language learning challenges.

## Highlights

• Questionnaires can be useful tools for obtaining information on auditory behavior.
• APDQ demonstrates separating the three clinical groups studied.
• The translated version of the questionnaire showed good reliability parameters.

## Introduction

Central Auditory Processing (CAP) refers to the functions of the Central Auditory Nervous System (CANS) which parses auditory information, and involves sound stimuli analysis and interpretation.^1^, ^2^

Central Auditory Processing Disorder (CAPD) has been recognized as a clinical entity since 2005,^2^ which means the condition has distinct and finite features with clearly referenced diagnostic criteria. The American Speech-Language-Hearing Association (ASHA) establishes that CAPD refers to difficulties in processing auditory information in the central nervous system and the neurobiological activity that underlies this process that generates electrophysiological auditory potentials.^2^

The behaviors observed in children with CAPD include hearing, school, and social difficulties.^3^, ^4^, ^5^, ^6^, ^7^, ^8^

Lately, there has been much controversy about the recognition of CAPD as a single clinical entity in the United States, especially with regards to overlapping clinical manifestations with other disorders, questions about whether it is primarily a top-down or bottom-up sensory-neural deficit and an inability of the scientific community to reach a consensus on diagnosis and intervention planning.^9^, ^10^, ^11^, ^12^

In any case, CAP diagnosis has been carried out through behavioral tests aiming to assess the auditory mechanisms and electrophysiological tests to evaluate the integrity of the central auditory pathway.^2^, ^3^

The international guidelines and consensus statements on CAP^2^, ^3^, ^9^, ^13^, ^14^, ^15^ recommend the use of questionnaires and scales to identify individuals at risk for CAPD, as they provide information about the individual’s communication deficits and the functional impact on the individual's communication, academic or work performance.

Several questionnaires that investigate hearing and listening skills have been developed and/or studied in the international literature, with excellent psychometric characteristics and with great potential to detect individuals who are likely to have CAPD.^11^, ^16^, ^17^, ^18^ Although questionnaires are used by 75% of educational audiologists in the United States to investigate issues related to CAPD,^19^ there are disagreements about the validity of the questionnaires, degrees of sensitivity and specificity for CAPD, difficulty in reading and interpreting the questions,^10^ and about not performing differential screening with other disorders, such as attention and language.

In Brazil, there is a shortage of questionnaires with methodological rigor in Brazilian–Portuguese language, intended to assist with the screening and clinical delineation of auditory processing disorders.^20^, ^21^

In order to develop an instrument to assist with the diagnosis of CAPD and differentiate children at risk for CAPD from those most likely to have Attention Deficit Hyperactivity Disorder (ADHD) or a language disorder, Brian O’Hara, in 2007, developed a questionnaire called The Auditory Processing Domains Questionnaire (APDQ) aiming at tracking individuals who may have CAPD due to listening difficulties they present. The APDQ assesses auditory abilities, hearing problems, language skills and some aspects of attention.

The APDQ instrument was created, presented and validated in the United States with 280 subjects in the state of Hawaii which included 198 normal controls.^22^ Sensitivity and specificity levels were above 80% in being able to correctly identify normal controls and the 3 clinical groups including APD, ADHD and language/learning challenges. It has been translated into several languages including Norwegian, Spanish, French, Turkish and Persian.^23^

Although it is one of the questionnaires recommended by the various guidelines and consensus statements on auditory processing,^15^, ^24^ few reports^22^, ^23^, ^25^ have studied the APDQ, and it has not been translated into Brazilian–Portuguese language.

The present study aimed to translate, adapt, and determine the APDQ validity and reliability in Brazilian–Portuguese language.

## Methods

This was a descriptive and exploratory study to validate the questionnaire. It was submitted and approved by the Ethics and Research Committee on Human Beings of the Federal University of Sao Paulo (UNIFESP) under number 27920214.8.0000.5505 and carried out in partnership with the Federal University of Santa Catarina (UFSC). The legal guardians of the selected students signed the Free and Informed Consent Form, agreeing to participate in the research, and the students signed the Term of Assent. The research started in 2014 but the final analysis was concluded in 2020.

## Participants

In the first phase of the questionnaire application, 10 children without communication complaints, aged 9–17 years participated. In the second phase, the questionnaire was applied to 66 students from the cities of São Paulo and Florianópolis, aged 7–17 years without any hearing, communication, oral or written language, memory or learning complaints. In addition, they did not present pre or perinatal health challenges, history of language delay, recurrent ear infections or other related problems.

The author of the APDQ questionnaire authorized the use of the questionnaire for this research (Appendix A).

### Materials

APDQ^22^ was translated and adapted into Brazilian–Portuguese language, according to the steps described below:

A The author of the questionnaire was contacted and approved the translation and use of APDQ in a Brazilian population.
B Initial translation: The APDQ’s items in the English version were initially translated into Brazilian Portuguese by two independent Brazilian bilingual speech therapists, aware of the purpose of this research. The two translations were compared by the translators and coordinators of the study, and, in case of disagreements, modifications were made until a consensus was reached on the initial translation (version 1 in Brazilian Portuguese) (Appendix B).
C Evaluation of the initial translation (back translation): The initial translation was translated into English by an American English teacher, and by another bilingual speech therapist, who did not participate in the previous stage. Subsequently, the two English versions were compared with the original instrument in English and the existing discrepancies were documented and analyzed. Thus, an English version of the APDQ translation was produced into Brazilian Portuguese. This English version was approved by the APDQ’s author and, in addition, he sent a 2014 updated version of the APDQ.
D After the approval of the translated version, version 1 in Brazilian Portuguese received some adjustments based on the author's updated version, and version 2 in Brazilian Portuguese was generated. To this end, a committee of experts in audiology and speech therapy performed the translation review and adjustments.
E Evaluation of cultural equivalence: The questionnaire in Brazilian Portuguese was applied to the parents of 10 students, without communication complaints. To each of the 52 questions in Brazilian Portuguese in its version 2, the option “not applicable” was added, to identify misunderstandings or irregularly performed issues by our population, and, therefore, considered culturally inappropriate. Questions rated over 15% for a “not applicable” answer would be selected and evaluated to be replaced by others of the same concept. However, no questions were answered as “not applicable”, and then, the final version of the questionnaire was achieved (Appendix C).

The student's performance was rated on each of the questions, using a 4-point scale. The scoring was performed as follows: four points if behavior is observed most times; three points if behavior is observed frequently; one point if behavior is observed sometimes and zero points if behavior is observed rarely.

The questionnaire consists of three domains: auditory processing (AP scale) with 31 items; attention (ATT scale) with 10 items (one overlapping with the AP scale) and language (LANG scale) with 11 items (one overlapping with the AP scale). The fourth scale is called Targeted Auditory Processing (TAP), consisting of 18 items of auditory decoding (questions 5, 8, 9, 10, 11, 12, 13, 21, 23, 27, 32, 34, 35, 36, 37, 49, 51 and 52), and used as a proxy of the auditory processing domain for research purposes or in cases where manual filling of the scoring spread sheet is necessary.

In this way, the maximum points that can be obtained in each domain are 124, 40 and 44, respectively for auditory processing, attention, and language (Table 1).

**Table 1:** Number of questionnaire items’ separated by domain.

Domains	Questions
Auditory Processing	3, 4, 5, 9, 10, 11, 12, 13, 15, 18, 21, 22, 23, 27, 28, 32, 33, 34, 35, 36, 37, 39, 40, 43, 44, 45, 46, 47, 49, 51 and 52
Attention	1, 3, 6, 14, 17, 20, 24, 29, 31 and 42
Language	7, 16, 19, 25, 26, 28, 30, 38, 41, 48 and 50
TAP	5, 8, 9, 10, 11, 12, 13, 21, 23, 27, 32, 34, 35, 36, 37, 49, 51 and 52

The performance scores presented in percentage (raw percentile) per domain, are calculated as follows:

$$\frac{total\;score\;obtained\;in\;the\;questions\;of\;the\;given\;domain}{4\;\times \;the\;maximum\;score\;in\;that\;domain}\times 100$$

**Figure 1:**
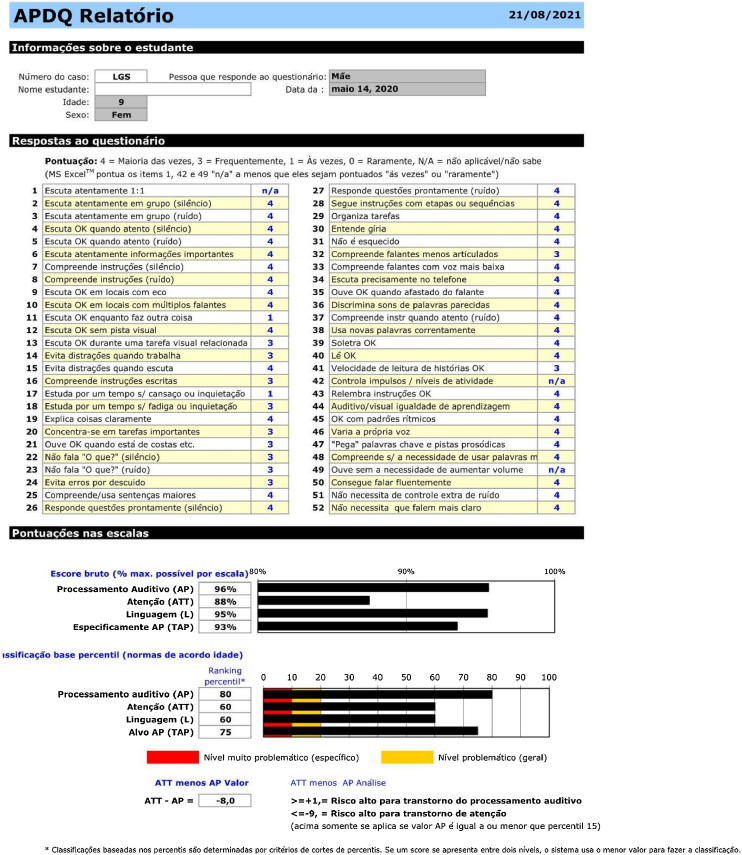
Excel file APDQ report form.

Therefore, after data collection, the answers were recorded in an Excel spreadsheet that calculates all scores in each domain and generates a score in percentage (raw percentile). A rank of this percentage according to a percentile (rank percentile) is also calculated, as shown in Fig. 1. For detailed information on the calculation of scores, we recommend reading the article the authors published the questionnaire.^22^

After a 15-day interval, the questionnaire was retested. The legal guardians of the 66 students who participated in the first phase were asked to rate the questionnaire again. It is noteworthy that the evaluator did not interfere in any of the stages.

### Statistics

The data from the present study were stored in Microsoft Excel spread sheets (2019) and the analyzes were performed using The Statistical Package for Social Sciences (SPSS) 25.0 software.

Categorical variables with their respective 95% Confidence Intervals (95% CI) and continuous variables were presented. Mann-Whitney *U* test was applied to assess differences between medians of the groups.

In the analysis of the instrument’s internal consistency, Cronbach’s Alpha coefficient was used.^26^, ^27^ For external reliability, Intraclass Correlation Coefficient (ICC) and Pearson’s correlation coefficient were used.^28^, ^29^ The significance level was set at 5%.

## Results

In order to develop the APDQ Brazilian–Portuguese version, in the first phase of this study, the APDQ questionnaire in its version 2 was applied to the parents of ten students aged 9–17 years old, seven female and three male. As all items in the questionnaire were fully understood, we considered that an acceptable version of the questionnaire had been achieved.

In the second phase, 66 students participated in the study, the majority of participants were female (62.1%), aged between 7 and 10 years old (56.1%) and enrolled in elementary school (78.8%). Regarding the interviewees, the mothers represented 89.4% of the respondents. In addition, regarding the schooling level, a greater proportion of parents and/or guardians (87.87%) reported having at least completed higher education (Table 2).

**Table 2:** Description of the sample characteristics (n = 66).

Variables	Total		
	n	%	95% CI
Gender			
Female	41	62.1	49.6–73.2
Male	25	37.9	26.7–50.3
Age group			
7–10 years	37	56.1	43.6–67.7
11–17 years	29	43.9	32.2–56.3
School grade			
1st to 4th	27	40.9	29.4–53.3
5th to 9th grade	25	37.9	26.7–50.3
10th to 12th grade	13	19.7	11.6–31.3
Higher education (enrolled)	1	1.5	0.2–10.4
Person completing questionnaire			
Father	4	6.1	2.2–15.4
Mother	59	89.4	79.1–94.9
Teacher	–	–	–
Other	3	4.5	1.4–13.2
Respondent’s education level			
Incomplete high school	–	–	–
Complete high school	7	10.6	5.0–20.9
Incomplete undergraduate course	1	1.5	1.2–10.4
Complete undergraduate course	37	56.1	43.6–67.7
Incomplete graduate course	4	6.1	2.2–15.4
Complete graduate course	17	25.7	16.4–37.9

n, number of participants; %, percentage; CI, Confidence Intervals.

When stratifying by age groups, the older group (11–17 years old) had the highest total medians scores and median percentage scores (raw score) in all domains when compared to the younger group, with a statistically significant difference in the auditory processing and attention domains (Table 3).

**Table 3:** Mean and medians total scores and scores in percentage (raw score) according to age groups and questionnaire domains.

Scores	7–10 years (n = 37)	11–17 years (n = 29)	*p*-Value^a^	Total
AP Mean (SD)	106.73 (13.10)	113.80 (10.31)	0.003^a^	109.70 (12.12)
Min–Max	56–120	79–120		56–120
AP Median	111.00	118.00		114.00
ATT Mean (SD)	26.00 (6.41)	28.75 (3.59)	0.047^a^	27.21 (5.51)
Min–Max	10–33	20–32		10–33
ATT Median	28.00	30.00		29.00
LANG Mean (SD)	41.47 (3.05)	42.48 (3.23)	0.084	41.91 (3.15)
Min–Max	33–44	28–44		28–44
LANG Median	43.00	44.00		43.00

Raw score	7–10 years (n = 37)	11–17 years (n = 29)	*p* value^a^	Total
AP Mean (SD)	88.78 (12.98)	94.17 (9.69)	0.004^a^	91.15 (11.87)
Min–Max	33–100	56–100		33–100
AP Median	93.00	98.00		96.00
ATT Mean (SD)	79.98 (19.73)	89.45 (11.96)	0.026^a^	84.14 (17.31)
Min–Max	31–100	61–100		31–100
ATT Median	88.00	94.00		89.00
Lang Mean (SD)	94.47 (6.91)	96.55 (7.35)	0.080	95.38 (7.13)
Min–Max	75–100	64–100		64–100
Lang Median	98.00	100.00		98.00
TAP Mean (SD)	87.84 (13.21)	94.13 (8.97)	0.004*	90.60 (11.88)
Min–Max	33–100	62–100		33–100
TAP Median	92.00	97.00		95.00

AP, Auditory Processing domain; ATT, attention domain; LANG, language domain; TAP, Targeted Auditory Processing; SD, standard deviation; Min, minimum; Max, maximum.

a *p*-Value for the Mann–Whitney *U* Test.

Table 4 shows the number of participants according to the scale score percentile rank (rank percentile), calculated by the original instrument. In both age groups, most individuals concentrated on the 90th percentile rank.

**Table 4:** Intraclass Correlation Coefficient (ICC) and Pearson’s Correlation (PC) for the questionnaire test and retest, according to the auditory processing, attention, and language domains (n = 25).

Domains	CCI			*p*	Pearson	*p*
	R	95% CI				
		Lower bound	Upper bound		Total R	
Auditoryprocessing	0.94	0.87	0.97	<0.001		
Attention	0.72	0.36	0.87	0.001		
Language	0.93	0.85	0.97	<0.001		
Total score	0.95	0.90	0.98	0.001	0.93	<0.001

CCI, Intraclass Correlation Coefficient; CI, Confidence Intervals; R, Pearson's correlation coefficient.

In the internal reliability of the questionnaire, Cronbach's alpha coefficient revealed reliable results for the auditory processing, attention and language domains, with the respective scores of 0.93, 0.85 and 0.74.

Twenty-five parents (37.87%) from this group re-rated their questionnaires after a fifteen-day interval so retest reliability could be checked. Table 4 shows the results of the measures which assessed the questionnaire's external reliability. The analysis performed through the intraclass coefficient showed excellent results for the auditory processing (R = 0.94) and language (R = 0.93) (*p* < 0.001) domains, and a substantial result for the attention domain (R = 0.72) (*p* = 0.001). Pearson's correlation calculated for the total score indicated strong test–retest reliability.

## Discussion

In relation to the 66 participants in this research, we found that the majority were female, representing almost a 2:1 ratio in relation to male participants. Although our study did not describe a representative population in terms of men-women proportion, other studies applying the APDQ did not find gender interference in the results of children without CAPD complaints.^22^, ^25^

The majority of the individuals who answered the questionnaire in the last step of cultural equivalence evaluation (87.87%) had at least completed higher education. This level of education corresponds to 12.5% of the Brazilian population in 2020.^30^ These data may have influenced the respondents' understanding of the questionnaire items, contributing to the good responses obtained and thus corroborating the expectation of having over 85% of the participants who had no difficulties understanding the questions.^31^

Stratification by age group was carried out according to the original publication of the APDQ questionnaire, which presents a total mean and medians score and a percentage score separated in the two age groups presented in this study,^22^ as age is a significant factor for the three questionnaire domains. The present study found that there was a significant difference as the age range increased for the total score and raw scores for the attention and auditory processing domains, this is in line with a study that applied the APDQ in school children aged eight to twelve years.^25^

It is noteworthy that student’s listening abilities in the younger age group are inferior to those over 11/12 years, which is the same age of peak performance on CAP tests and presumed Central Auditory Nervous System maturity peak. This means that the responses in this older age group are similar to those obtained in adult individuals^32^, ^33^ and, therefore, the battery of behavioral tests to identify CAPD must consider the age of the children to be assessed.^34^, ^35^ Studies also show that the neural substrate related to attention also changes with age,^36^ demonstrating that the attention level of children increases with age.^37^, ^38^ As the child develops and faces greater demands, significant changes occur in information processing and attentional skills, which leads to an increase in attentional capacity, pointing to permanent neuronal development.^39^, ^40^, ^41^ Neuropsychological tests also present different criteria for each age group in the attention domain.

The auditory processing and attention scales means, and SD obtained in this study (Table 3) are slightly above those in the original US study.^22^, ^23^ In this study, the attention domain presented the lowest mean scores, corroborating the findings of the original U.S. study. In the Persian APDQ validation study with 8–12 y/o students, the scores for all domains were very similar but with normal controls having slightly lower scores in the language domain differing from the findings of the original U.S. and our present study.^25^

According to the original U.S. study, 7- to 10-year-old children with percent perfect scale scores below 68%, 61% and 79% for auditory processing, attention and language, respectively, are considered to be at moderate clinical risk.^22^ These findings were confirmed in the Persian study that applied the APDQ to school children with and without suspected CAPD. Children with suspected CAPD, according to the results of the APDQ, had significantly lower scores compared to normal controls. Mean scores for auditory processing, attention and language domains were respectively: 67, 61 and 64.^25^ This screening outcome was later confirmed by sub-normal behavioral auditory test scores including dichotic digits tests and speech in noise suggestive of CAPD.

**Figure 2:**
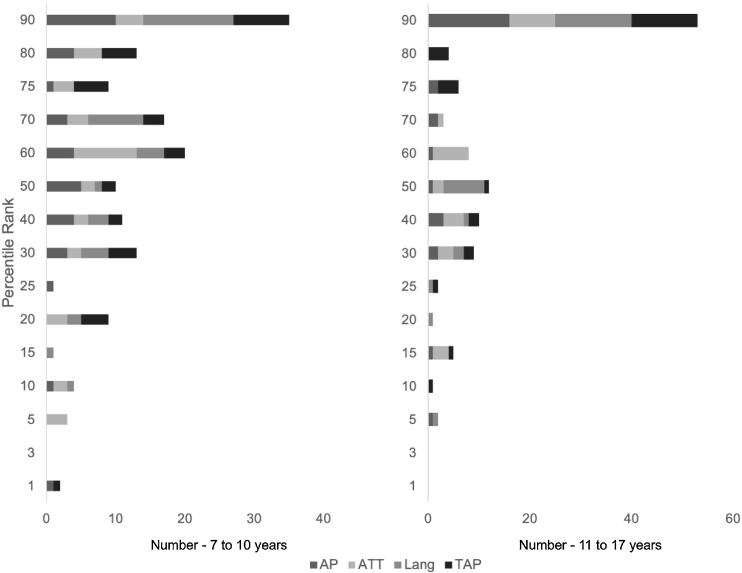
Number of children according to percentile, age group and questionnaire domain. Auditory Processing (AP scale); Attention (ATT scale); Language (LANG scale); TAP (Targeted Auditory Processing).

For participants aged 11–17 years, scale scores below 72%, 69% and 82% for auditory processing, attention and language are considered to be at risk and should be referred for diagnostic tests.^22^ Most of the students in this research were classified in the 90th percentile (Fig. 2). It is worth mentioning that only individuals who did not present any risk factor were included based on the items listed on the first page of the APDQ questionnaire, which certainly contributed to the formation of a group with typical development and without evidence of CAPD or other clinical diagnoses.

CAPD frequently occurs with other learning or developmental disorders, with language and literacy impairment as the most frequent.^3^, ^6^, ^15^ The APDQ questionnaire demonstrated its ability to differentiate between three clinical groups: those with auditory processing disorder, attention deficit disorder and learning disorders, with sensitivity and specificity values over 80%, according to data obtained in the author’s publication on the questionnaire^22^ and in others studies.^25^ Hence, the APDQ can be a valuable tool when approaching the child with hearing and listening complaints. It is possible that some of these children will meet screening criteria for attention or learning risk factors. The data obtained with the APDQ application may suggest that other assessments should be carried out.^25^

Cronbach's Alpha Coefficient, which evaluated the internal consistency of the questionnaire responses, showed a strong correlation for all domains. The reliability of the questionnaire items obtained in this study are similar to those found by the authors of the original study,^22^ which presented 0.96 for the auditory processing domain, and 0.88 for the attention and language domains, in line with the Persian study domains, which presented the measures of 0.92, 0.86 and 0.88 for the auditory processing, attention and language domains, respectively.^23^

Likewise, the analysis of external reliability was adequate, showing that there were no significant changes in the test-retest responses. In the other cultural adaptation studies, the measures were found to be very similar to the present study (Table 4), reinforcing the high probability of obtaining the same results if the instrument is applied again to the same population.

In a systematic review,^21^ it was found a shortage of studied instruments validated for Brazilian–Portuguese language which obey the rules previously established for the translation and validation of questionnaires.^16^, ^42^, ^43^, ^44^, ^45^ In this context, this study is believed to contribute to expand the possibility of using questionnaires in CAPD evaluations enabling its use in clinical practice and in research.

The information obtained through well-structured questionnaires on the child's auditory behavior can be very helpful in the early identification of auditory information processing discrepancies, which then points the way to appropriate diagnostic evaluations and treatment opportunities. However, we highlight it should not be performed as the only form of diagnosis. In addition, we recommend further research using the APDQ in different populations with clinical diagnoses and control groups be carried out.

Recently, the questionnaire has undergone minor modifications, in which the questions have been revised and updated and a new version of the questionnaire with 50 questions made available by Education Audiology Association online store in Pittsburgh Pennsylvania in conjunction with the scoring software or user manual. In this way, research is being carried out in different populations in order to continue the studies involving the screening instrument.

In this study, a Brazilian Portuguese version of the APDQ questionnaire was obtained through a translation and validation process. The next step includes the APDQ application in groups of children with and without auditory processing disorder, attention deficits and language learning challenges.

Finally, further research is needed to explore the performance of the questionnaire in different pathological groups, as well as in adult individuals. Furthermore, we suggest the application of the instrument to individuals with different socioeconomic and educational levels.

## Conclusion

According to the results of this study, the Brazilian–Portuguese version of the APDQ has excellent parameters for translation, adaptation, validation, and reliability.

## Conflicts of interest

The authors declare no conflicts of interest.

## Figures and Tables

**Figure:**
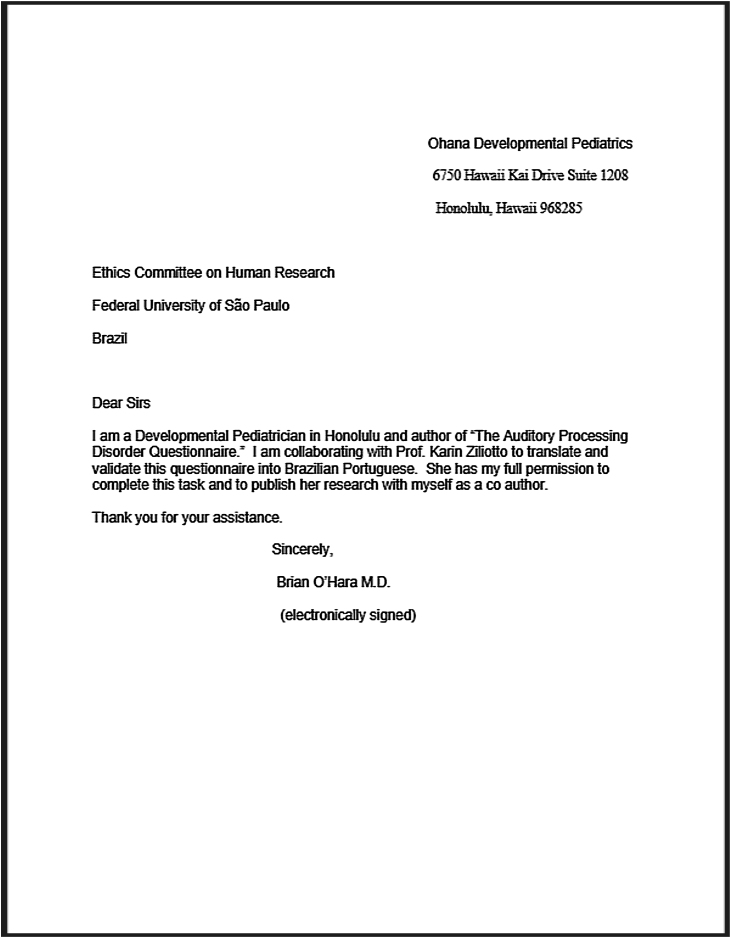


**Table:** 

Questionário dos Domínios do Processamento Auditivo
Para pais e professores de estudantes de 7 a 17 anos.
Instruções:
Este questionário revisa as habilidades auditivas do dia a dia de um estudante. Linguagem, atenção e habilidades auditivas são importantes.
Favor avaliar o desempenho do estudante em cada um dos itens abaixo baseado em suas observações. Lembre-se do que é esperado para ele ou ela na sua idade. O termo “ambientes ruidosos” refere-se aos ruídos de fundo de TV, vozes, música, máquinas, etc. Ruídos leves a moderados podem interferir na habilidade de ouvir palavras corretamente. “Ouvir corretamente” significa ouvir as declarações corretamente, sem precisar de repetições.
Assinale:
Coluna 1: se a habilidade for observada regularmente (mais de 75%)
Coluna 2: se a habilidade for observada frequentemente (mais de 50%)
Coluna 3: se a habilidade for observada algumas vezes (menos de 50%)
Coluna 4: se a habilidade for observada raramente (menos de 25%)
Avalie todos os itens – escreva N/A se for incapaz.

**Table:** 

	Quase sempre (mais de 75%)	Frequen-temente (mais de 50%)	Às vezes (menos de 50%)	Raramente (menos de 25%)
1. Presta bem atenção quando conversa com uma única pessoa.				
2. Presta bem atenção ao ouvir em ambientes silenciosos na presença de outras pessoas (refeições, reuniões, aulas etc.).				
3. Presta bem atenção ao ouvir em ambientes ruidosos na presença de outras pessoas (refeições, reuniões, aulas, etc).				
4. Não tem dificuldade em ouvir o que você fala – ouve corretamente quando presta bastante atenção em ambientes silenciosos.				
5. Não tem dificuldade em ouvir o que você fala – ouve corretamente quando presta bastante atenção em ambientes ruidosos.				
6. Dedica um tempo para ouvir cuidadosamente e corretamente uma informação importante.				
7. Compreende instruções faladas quando o ambiente está silencioso.				
8. Compreende instruções faladas quando o ambiente está ruidoso.				
9. Compreende os outros quando conversa em locais cheios e com eco – academias, refeitórios, shoppings.				
10. Consegue entender a sua conversa enquanto outra acontece ao lado (em festas, refeições e outras reuniões).				
11. Se interessado, consegue te ouvir corretamente enquanto faz outra coisa (vídeo-games, pequenas tarefas, assistindo TV etc.).				
12. Consegue ouvir corretamente e atentamente sem pistas visuais (sem ver a face, gestos, imagens do falante etc.).				
13. Consegue ouvir corretamente enquanto realiza uma tarefa visual relacionada - verificando uma página ou o quadro-negro e tomando notas (se for mais velho).				
14. Concentra-se bem – ignora distrações quando faz atividades que não exigem ouvir (estudar, outras tarefas).				
15. Concentra-se bem – ignora distrações quando ouve histórias e apresentações.				
16. Compreende instruções escritas (conforme esperado para a idade).				
17. Não se cansa facilmente quando estuda por longos períodos – (boceja ou brinca com as mãos - considere idade).				
18. Não se cansa facilmente quando escuta por longos períodos (boceja ou brinca com as mãos – considere idade).				
19. Consegue explicar coisas razoavelmente bem durante conversas.				
20. Concentra-se em coisas importantes – completa tarefas mesmo que não sejam urgentes ou muito interessantes (para ele/ela).				
21. Ouve bem as palavras quando o falante está de costas ou está atrás dele/dela.				
22. Não fala “ãhn?”, “o quê?” ou necessita repetições quando conversa com interesse em ambientes silenciosos.				
23. Não fala “ãhn?”, “o quê?” ou necessita repetições quando conversa com interesse em ambientes ruidosos.				
24. Presta atenção a detalhes – evita erros por descuido quando faz a tarefa escolar.				
25. Compreende e usa frases mais longas e complexas (conforme esperado para a idade).				
26. Compreende e responde prontamente a perguntas em ambientes silenciosos (quando atento).				
27. Compreende e responde prontamente a perguntas em ambientes ruidosos (quando atento).				
28. Consegue seguir instruções orais mais complexas, com etapas ou sequências (conforme esperado para a idade).				
29. Organiza tarefas e atividades para realizá-las de uma maneira ordenada e em tempo (conforme esperado para a idade).				
30. Compreende e usa gírias comuns para sua idade (sou fera, é da hora, etc).				
31. Não perde ou esquece de fazer as atividades do dia-a-dia (não é avoado).				
32. Compreende pessoas que falam palavras de forma menos clara (rápido ou enrolado, com sotaques etc.).				
33. Compreende de vozes suaves a altas dos falantes.				
34. Ouve bem ao telefone sem precisar de repetição (incluindo nomes e números).				
35. Consegue ouvir as pessoas (e a televisão) corretamente a uma distância de 2 metros, aproximadamente.				
36. Não ouve errado nem confunde palavras com som parecido em ambientes ruidosos (como faca e vaca, sessenta e setenta).				
37. Compreende instruções em ambientes ruidosos quando presta bastante atenção ao falante.				
38. Usa novas palavras corretamente logo depois de aprendê-las (conforme esperado para a idade).				
39. Consegue emitir os sons que formam uma palavra para ajudar na soletração ou corrigir erros de soletração: (conforme esperado para a idade).				
40. Consegue emitir os sons que formam uma palavra e falar palavras não-familiares corretamente quando está aprendendo a lê-las (conforme esperado para a idade).				
41. Consegue ler e compreender histórias em uma boa velocidade (conforme esperado para a idade)				
42. Controla prontamente impulsos e agitação a fim de evitar situações perigosas e que podem aborrecer.				
43. Lembra de detalhes de instruções ou pedidos feitos verbalmente, pouco tempo depois, sem a necessidade de repetição.				
44. Aprende bem as coisas ouvindo – sem precisar muito de mais explicações ou de apoio visual.				
45. Segue prontamente padrões rítmicos e de entonação de fala ao recitar versos, reproduzir uma música batendo palmas ou cantarolando, etc.				
46. Varia a própria voz para dar ênfase, falar com clareza e parecer mais agradável.				
47. Interpreta comentários e segue instruções prontamente levando em consideração “como” as pessoas falam (percebe diferentes tons de voz, ênfase em palavras chaves etc.).				
48. Compreende o que é falado sem precisar de palavras mais simples (conforme esperado para a idade).				
49. Ouve bem sem precisar aumentar o volume das coisas (aumentar o volume da televisão, sentar-se mais perto ou pedir que falem mais alto).				
50. Consegue falar relativamente rápido e sem problemas, para a idade (sem uso de “ãhn” ou pausas para encontrar palavras e ideias).				
51. Ouve bem as pessoas sem precisar controlar tanto os ruídos ambientais (presta atenção mesmo sem ter que desligar o rádio, fechar janelas, trocar de lugar etc.).				
52. Não é necessário que falem mais devagar e claramente para ajudá-lo a ouvir corretamente.				

**Table:** 

INFORMAÇÕES PESSOAIS								
1. Data: ____/____/____								
2. Idade do estudante: ______anos e ______ meses								
4. Escolaridade: _____								
5. Pessoa que preenche o questionário:								
1. Data: ____/____/____								
( ) pai	( ) mãe	( ) professor(a)	( ) outro					
6. Raça do estudante (pode marcar mais de um, porém circule o principal, se possível):								
( ) branca	( ) preta	( ) amarela	( ) indígena	( ) parda	( ) sem declaração			
7. Escolaridade do pai: __________________								
8. Escolaridade da mãe: _________________								
9. Assinale o grau da sua preocupação com as habilidades auditivas do estudante:								
( ) nenhuma	( ) leve	( ) moderada	( ) alta					
10. Assinale quando uma ou mais das condições ou serviços abaixo ocorreram para o estudante:								
a. ( ) Educação especial								
b. ( ) Dificuldade de aprendizagem								
c. ( ) Dislexia (ou Distúrbio de Aquisição da Linguagem)								
d. ( ) Histórico de atraso de fala/linguagem ou de terapia fonoaudiológica								
e. ( ) Perda Auditiva permanente								
( ) Usa prótese auditiva	( ) Não usa prótese auditiva	- ( ) Unilateral	( ) Bilateral	- ( ) Leve	( ) Moderada	( ) Severa	( ) Profunda	f. ( ) Aprendeu o português como segunda língua após os 5 anos de idade
g. ( ) Transtorno do Déficit de Atenção e Hiperatividade								
Toma medicamento:	( ) sim	( ) não	h. ( ) Otites médias crônicas ou cirurgias no ouvido.					
i. ( ) Icterícia ao nascimento								
( ) leve	( ) moderada	( ) severo						
j. ( ) Distúrbio do Processamento Auditivo								
k. ( ) Autismo/Síndrome de Asperger								
l. ( ) Atraso do desenvolvimento (retardo mental)								
m. ( ) Nenhuma das alternativas								

**Table:** 

Questionário dos Domínios do Processamento Auditivo
Para pais e professores de estudantes de 7 a 17 anos.
Instruções:
Este questionário revisa as habilidades auditivas do dia a dia de um estudante. Linguagem, atenção e habilidades auditivas são importantes.
Favor avaliar o desempenho do estudante em cada um dos itens abaixo baseado em suas observações. Lembre-se do que é esperado para ele ou ela na sua idade. O termo “ambientes ruidosos” refere-se aos ruídos de fundo de TV, vozes, música, máquinas etc. Ruídos leves a moderados podem interferir na habilidade de ouvir palavras corretamente. “Ouvir corretamente” significa ouvir as declarações corretamente, sem precisar de repetições.
Assinale:
Coluna 1: se a habilidade for observada regularmente (mais de 75%)
Coluna 2: se a habilidade for observada frequentemente (mais de 50%)
Coluna 3: se a habilidade for observada algumas vezes (menos de 50%)
Coluna 4: se a habilidade for observada raramente (menos de 25%)
Avalie todos os itens – escreva N/A se for incapaz.

**Table:** 

	Quase sempre (mais de 75%)	Frequen-temente (mais de 50%)	Às vezes (menos de 50%)	Rara-mente (menos de 25%)
1. Presta bem atenção quando conversa com uma única pessoa.				
2. Presta bem atenção ao ouvir em ambientes silenciosos na presença de outras pessoas (refeições, reuniões, aulas, etc).				
3. Presta bem atenção ao ouvir em ambientes ruidosos na presença de outras pessoas (refeições, reuniões, aulas, etc).				
4. Não tem dificuldade em ouvir suas palavras corretamente, quando presta bastante atenção em ambientes silenciosos.				
5. Não tem dificuldade em ouvir suas palavras corretamente, quando presta bastante atenção em ambientes ruidosos.				
6. Dedica um tempo para ouvir cuidadosamente e corretamente uma informação importante.				
7. Compreende instruções faladas quando o ambiente está silencioso.				
8. Compreende instruções faladas quando o ambiente está ruidoso.				
9. Compreende os outros quando está em locais grandes com eco - academias, refeitórios, shoppings.				
10. Consegue entender a *sua* conversa enquanto outra acontece ao lado (em festas, refeições e outras reuniões).				
11. Se interessado, consegue te ouvir corretamente enquanto faz outra coisa (vídeo-games, pequenas tarefas, assistindo TV, etc).				
12. Consegue ouvir corretamente sem pistas visuais (sem ver a face, gestos, imagens do falante, etc).				
13. Consegue, ao mesmo tempo, olhar e ouvir corretamente - verificar uma página ou o quadro-negro e tomar notas (se for mais velho).				
14. Concentra-se bem quando faz atividades que não exigem ouvir (estudar, outras tarefas).				
15. Concentra-se bem quando ouve histórias e apresentações.				
16. Compreende instruções escritas (conforme esperado para a idade).				
17. Não se cansa facilmente quando estuda – (boceja ou brinca com as mãos - considere idade).				
18. Não se cansa facilmente quando escuta (boceja ou brinca com as mãos – considere idade).				
19. Consegue explicar coisas razoavelmente bem durante conversas.				
20. Concentra-se em tarefas mesmo que não sejam urgentes ou muito interessantes (para ele/ela).				
21. Ouve bem as palavras quando o falante está de costas ou está atrás dele/dela.				
22. Não fala “ãhn?”, “o quê?” ou necessita repetições quando conversa com interesse em ambientes silenciosos.				
23. Não fala “ãhn?”, “o quê?” ou necessita repetições quando conversa com interesse em ambientes ruidosos.				
24. Presta atenção a detalhes – evita erros por descuido quando faz a tarefa escolar.				
25. Compreende e usa frases mais longas e complexas (conforme esperado para a idade).				
26. Compreende e responde prontamente a perguntas em ambientes silenciosos (quando atento).				
27. Compreende e responde prontamente a perguntas em ambientes ruidosos (quando atento).				
28. Consegue seguir instruções orais, com etapas ou sequências (conforme esperado para a idade).				
29. Organiza tarefas e atividades para realizá-las em tempo (conforme esperado para a idade).				
30. Compreende e usa gírias comuns para sua idade (sou fera, é da hora, etc).				
31. Não perde ou esquece de fazer as atividades do dia-a-dia (não é avoado).				
32. Compreende pessoas que falam palavras de forma menos clara (rápido ou enrolado, com sotaques, etc).				
33. Compreende vozes suaves ou altas dos falantes.				
34. Ouve bem ao telefone sem precisar que a informação seja repetida (incluindo nomes e números).				
35. Consegue ouvir as pessoas (e a televisão) corretamente a uma distância de 2 metros, aproximadamente.				
36. Não ouve errado nem confunde palavras com som parecido (como faca e vaca, sessenta e setenta).				
37. Compreende instruções em ambientes ruidosos quando presta bastante atenção ao falante.				
38. Lembra e usa novas palavras corretamente (conforme esperado para a idade).				
39. Consegue emitir corretamente os sons que formam uma palavra para ajudar na soletração (conforme esperado para a idade).				
40. Consegue emitir os sons que formam uma palavra e falar palavras não-familiares corretamente quando está aprendendo a lê-las (conforme esperado para a idade).				
41. Consegue ler e compreender histórias em uma boa velocidade (conforme esperado para a idade)				
42. Controla prontamente impulsos e agitação a fim de evitar situações perigosas e que podem aborrecer.				
43. Lembra de detalhes de instruções ou pedidos feitos verbalmente, pouco tempo depois, sem a necessidade de repetição.				
44. Aprende bem as coisas ouvindo – sem precisar muito de mais explicações ou de apoio visual.				
45. Segue prontamente padrões rítmicos e de entonação ao reproduzir uma música batendo palmas ou cantarolando, etc.				
46. Varia a própria voz para dar ênfase, falar com clareza e parecer mais agradável.				
47. Reconhece “como” as coisas foram ditas ao interpretar comentários e seguir instruções (percebe diferentes tons de voz, ênfase em palavras chaves, etc).				
48. Compreende o que é falado sem precisar de palavras mais simples (conforme esperado para a idade).				
49. Ouve bem sem precisar aumentar o volume das coisas (aumentar o volume da televisão, sentar mais perto ou pedir que falem mais alto).				
50. Consegue falar facilmente e sem problemas, para a idade (sem uso de “ãhn” ou pausas para encontrar palavras e ideias).				
51. Ouve bem as pessoas sem precisar controlar os ruídos “extras” (presta atenção mesmo sem ter que desligar o rádio ou máquinas, fechar janelas, trocar de lugar, etc).				
52. Há pouca necessidade de que falem mais devagar e claramente para ajudá-lo a ouvir corretamente.				

**Table:** 

INFORMAÇÕES PESSOAIS							
1. Data: ____/____/____							
2. Idade do estudante: ______anos e ______ meses							
3. Sexo:______	4. Escolaridade:______________						
5. Pessoa que preenche o questionário:							
( ) pai	( ) mãe	( ) professor(a)	( ) outro				
6. Raça do estudante (pode marcar mais de um, porém circule o principal, se possível):							
( ) branca	( ) preta	( ) amarela	( ) indígena	( ) parda	( ) sem declaração		
7. Escolaridade do pai: __________________							
8. Escolaridade da mãe: _________________							
9. Assinale o grau da sua preocupação com as habilidades auditivas do estudante:							
( ) nenhuma	( ) leve	( ) moderada	( ) alta				
10. Assinale quando uma ou mais das condições ou serviços abaixo ocorreram para o estudante:							
a. ( ) Educação especial							
b. ( ) Dificuldade de aprendizagem							
c. ( ) Dislexia (ou Distúrbio de Aquisição da Linguagem)							
d. ( ) Histórico de atraso de fala/linguagem ou de terapia fonoaudiológica							
e. ( ) Perda Auditiva permanente							
( ) usa prótese auditiva	( ) não usa prótese auditiva	- ( ) unilateral	( ) bilateral	( ) leve	( ) moderada	( ) severa	( ) profunda
f. ( ) Aprendeu o português como segunda língua após os 5 anos de idade							
g. ( ) Transtorno do Déficit de Atenção e Hiperatividade							
Toma medicamento:	( ) sim	( ) não					
h. ( ) Otites médias crônicas ou cirurgias no ouvido.							
i. ( ) Icterícia ao nascimento							
( ) Leve	( ) Moderada	( ) Severo					
j. ( ) Distúrbio do Processamento Auditivo							
k. ( ) Autismo/Síndrome de Asperger							
l. ( ) Atraso do desenvolvimento (retardo mental)							
m. ( ) Nenhuma das alternativas							
